# mTOR kinase-dependent, but raptor-independent regulation of downstream signaling is important for cell cycle exit and myogenic differentiation

**DOI:** 10.4161/15384101.2014.941747

**Published:** 2014-10-30

**Authors:** Hilary J Pollard, Mark Willett, Simon J Morley

**Affiliations:** Department of Biochemistry, School of Life Sciences; University of Sussex; Brighton, UK; † Center for Biological Sciences; Faculty of Natural & Environmental Sciences; University of Southampton; Highfield Campus; Southampton, UK

**Keywords:** initiation factor, C2C12, translation, myoblasts, 4E-BP1, signaling, raptor

## Abstract

Myogenic differentiation in the C2C12 myoblast model system reflects a concerted and controlled activation of transcription and translation following the exit of cells from the cell cycle. Previously we have shown that the mTORC1 signaling inhibitor, RAD001, decreased protein synthesis rates, delayed C2C12 myoblast differentiation, decreased p70S6K activity but did not affect the hypermodification of 4E-BP1. Here we have further investigated the modification of 4E-BP1 during the early phase of differentiation as cells exit the cell cycle, using inhibitors to target mTOR kinase and siRNAs to ablate the expression of raptor and rictor. As predicted, inhibition of mTOR kinase activity prevented p70S6K, 4E-BP1 phosphorylation and was associated with an inhibition of myogenic differentiation. Surprisingly, extensive depletion of raptor did not affect p70S6K or 4E-BP1 phosphorylation, but promoted an increase in mTORC2 activity (as evidenced by increased Akt Ser473 phosphorylation). These data suggest that an mTOR kinase-dependent, but raptor-independent regulation of downstream signaling is important for myogenic differentiation.

## Introduction

The differentiation of skeletal muscle cells involves the exit of mononucleated myoblasts from the cell cycle, changes in activity of an array of signaling pathways, altered gene expression, and cell fusion to form multinucleated myotubes.^1,2^ It is likely that this process reflects the induction of IGF-II^3,4^ which signals via the Insulin-like growth factor 1 (IGF-1) receptor to activate phosphatidylinositol-3 kinase (PI3-K), protein kinase B (Akt), the Tuberous Sclerosis Complex (TSC1/2), and mammalian target of rapamycin (mTORC1) signaling.^5-7^ Activation of mTORC1 and phosphorylation of its downstream targets are central to the control of protein synthesis in many cell types, but the role of mTOR activity and its downstream signaling pathways in regulating differentiation in C2C12 myoblasts remains unclear.^8-13^

mTOR is contained within 2 distinct complexes: mTORC1 and mTORC2.^13-15^ The former complex includes mTOR, raptor, mLST8, proline-rich Akt substrate 40 kDa (PRAS40), DEP-domain-containing partner of mTOR (DEPTOR), and scaffolds tti1/tel2^7,13,16^ mTORC1 regulates the phosphorylation and activity of eIF4E-binding proteins (4E-BP1^5,7,14^) and the ribosomal protein S6 kinase, p70S6K.^17^ mTORC2, a complex including mTOR, rictor, protor1/2, DEPTOR, mLST8, and scaffolds tti1/tel2/mSin1 (reviewed in^7^), is known to regulate Akt, protein kinase Cα and serum-and glucocorticoid-induced protein kinase 1.^18-20^ In addition, mTORC2 plays an important role in regulating the actin cytoskeleton.^21^ Down-regulation of rictor has been reported to inhibit the ability of mTORC2 to phosphorylate Akt on Ser473 and stimulates protein synthesis in C2C12 myocytes.^12^

Protein synthesis is carried out in 3 stages (initiation, elongation and termination), with the initiation stage of translation generally accepted as a major site of regulation of gene expression in mammalian cells.^5,7,22-30^ This step in protein synthesis is regulated by a family of proteins, the initiation factors^5,23,24^ which interact with each other and the mRNA. These proteins modulate the binding of mRNA to the ribosome, a process facilitated by the assembly of the cap binding protein, eIF4E, a helicase, eIF4A, and a scaffold protein, eIF4G, into the eIF4F complex (eIF4E/eIF4A/eIF4G). The control of assembly of this complex is often dysregulated in tumor cells.^5-7,13,17,23^ The eIF4G scaffold protein possesses domains that interact with eIF4E, eIF4A, the multi-factor protein, eIF3, poly(A) binding protein (PABP) and the kinases, Mnk1/2, which modulate the phosphorylation of eIF4E on Ser209.^5,14,17,22–25^

The activity of the eIF4F complex is regulated its assembly, phosphorylation of components and the inherent structural properties of the recruited mRNA.^5,17,23-25^ Using a conserved motif, 4E-BP1 competes with eIF4G for a common surface on eIF4E and inhibits eIF4F assembly. Activation of mTORC1 leads to the multi-site phosphorylation of 4E-BP^14,15^ and the recruitment of mTORC1 to eIF3 resulting in p70S6K activation.^17,25^ The former prevents 4E-BP1 from binding to eIF4E and thereby allows formation of the eIF4F initiation complex and ribosomal recruitment of mRNA.^22-24^

Previously, we have shown that myogenic differentiation is associated with increased rates of translation, biphasic activation of p70S6K, and the phosphorylation of both eIF4E and 4E-BP1.^26^ Paradoxically, treatment of C2C12 myoblasts with an inhibitor of mTORC1 (RAD001^27^), delayed differentiation but led to the hypermodification of 4E-BP1 and to enhanced levels of initiation factor 4E (eIF4E)/4E-BP1 complex.^26^ Here we further investigate the signaling pathways responsible for the modification of 4E-BP1 during exit from the cell cycle using inhibitors of mTOR kinase and siRNAs to ablate expression of raptor and rictor. We show that as cells exit the cell cycle, depletion of raptor does not affect p70S6K or 4E-BP1 phosphorylation, but promoted an increase in mTORC2 activity (as evidenced by increased Akt Ser473 phosphorylation). mTOR activity is required for activation of p70S6K during myogenic differentiation but 4E-BP1 phosphorylation is insensitive to depletion of either raptor or rictor. These data suggest that an mTOR kinase-dependent, but raptor-independent regulation of downstream signaling is important for myogenic differentiation in mouse myoblasts.

## Results

### RAD001 inhibits p70S6K but maintains the phosphorylation of 4E-BP1 during myogenic differentiation

We and others have previously shown that rapamycin^10,12^ or RAD001^26^ potently inhibits myogenic differentiation in the C2C12 myoblast model system. Furthermore, we showed that although inhibition of mTORC1 reduced p70S6K activity, prevented the phosphorylation of rpS6, reduced protein synthesis rates and delayed myotube formation, paradoxically 4E-BP1 remained in a hyperphosphorylated form.^26^ To examine this further we have explored the phosphorylation of 4E-BP1 in more detail and compared the effects of RAD001 with kinase inhibitors of mTOR, such as Torin 1^13^ and KU0063794,^31^ and downstream signaling following ablation of rictor or raptor using siRNA.

**Figure 1.**
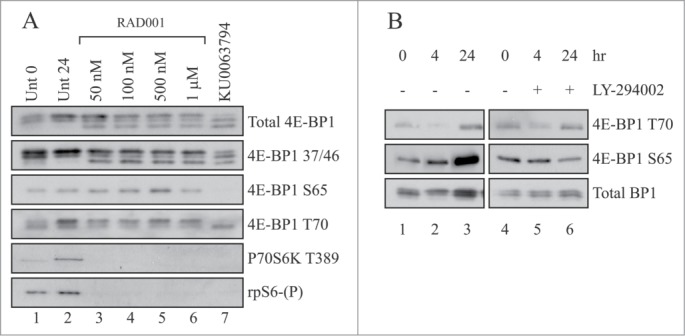
RAD001 inhibits p70S6K but maintains the phosphorylation of 4E-BP1 during myogenic differentiation. (**A**) C2C12 cells were grown to confluency and induced to differentiate by serum withdrawal in the presence of 2% (v/v) horse serum, 10 µg/ml insulin and 10 µg/ml transferrin (DM). Cells were harvested immediately (lane 1) or incubated for 24 hours in the absence (lanes 2) or presence of the indicated concentration of RAD001 (lanes 3–6) or 10 µM KU0063794 (lane 7). Extracts were prepared as described in Materials and Methods and aliquots containing 10 µg total protein resolved by SDS-PAGE and proteins visualized by Western blotting using the antisera shown. (**B**) Cells were incubated as above but in the absence or presence of 10 µM LY294002. Extracts were prepared as described in Materials and Methods and aliquots containing 10 µg total protein resolved by SDS-PAGE and proteins visualized by Western blotting using the antisera shown. In both cases, results are shown from a single experiment but similar data were obtained in at least 3 independent experiments.

**Figure 1A** shows that even when used at 1 μM levels for 24 hours, RAD001 did not totally prevent the phosphorylation of 4E-BP1 on Thr37/46, Ser65 or Thr70 (lane 6 vs. lanes 2–5). In contrast, relative to untreated cells (lanes 1 and 2), RAD001 even at 50 nM levels inhibited the phosphorylation of p70S6K and rpS6 (lane 3). To see if this was also true for other mTORC inhibitors, we used KU0063794 which inhibits the kinase activity of mTOR.^31^ **Figure 1A** shows that KU0063794 inhibits the phosphorylation of 4E-BP1 on Ser65, p70S6K and rpS6, while reducing the phosphoryation of Ser70 and Thr37/46 (lane 7 vs. lane 2). A partial decrease in 4E-BP1 phosphorylation was also seen when cells were induced to differentiate in the presence of the phosphatidylinositol 3-kinase (PI3-K) inhibitor, LY294002 (**Fig. 1B**). Under these conditions, LY294002 potently inhibited myogenic differentiation and eventually induced apoptosis in C2C12 myoblasts (data not shown).

**Figure 2.**
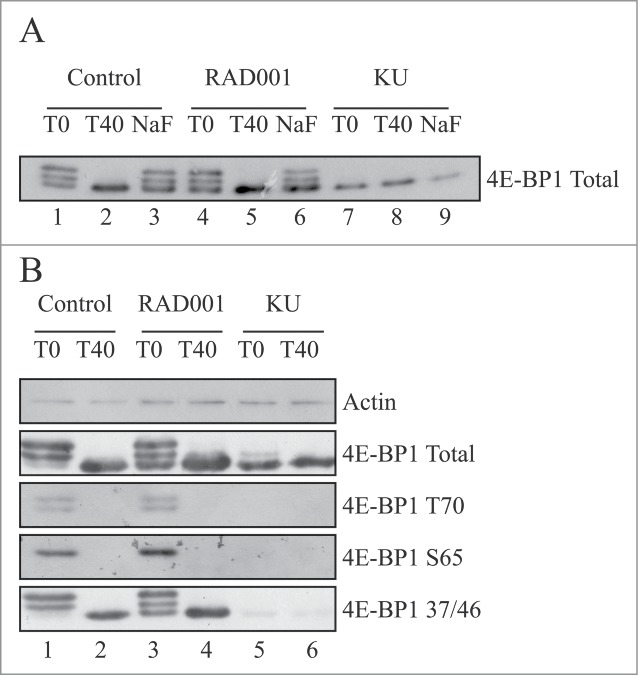
Phosphorylation of 4E-BP1 from RAD001-treated cells is sensitive to lambda phosphatase in vitro. (**A**) Cells were grown to confluency and induced to differentiate by serum withdrawal for 48 hours as in Figure 1, in the absence (control; lanes 1–3) or presence of 100 nM RAD001 (lanes 4–6) or 10 µM KU0063794 (lanes 7–9). Cell extracts were prepared and aliquots containing 90 µg total protein incubated with 200 units of lambda phosphatase for the times shown in the absence (lanes 1,2,4,5,7,8) or presence of 50 mM NaF (lanes 3,6,9) as described in Materials and Methods. Aliquots containing 10 µg total protein were resolved by SDS-PAGE and total 4E-BP1 protein visualized by Western blotting. (**B**) Cells were incubated as described above. Extracts were prepared and treated with lambda phosphatase as described in Panel A prior to SDS-PAGE and Western blotting using the antisera shown. Results are shown from a single experiment but similar data were obtained in at least 3 independent experiments.

Western blotting with phospho-specific antisera strongly suggests that even in the presence of RAD001, the phosphorylation of 4E-BP1 was maintained during differentiation. To address this more directly, we have used extracts prepared from cells incubated in the absence or presence of RAD001 or KU0063794 for 24 or 48 hours and subjected them to lambda phosphatase treatment. **Figure S1** shows that in differentiated cell extracts, this phosphatase was effective within 10 minutes, removing modification on Ser 65 more efficiently than Thr70 (lane 3 vs. lanes 2 and 1); Thr37/46 remained resistant to dephosphorylation (lanes 4–6) under these assay conditions. Similar results were seen with extracts from cells allowed to differentiate in the presence of RAD001 (lanes 8–13). **Figure 2A** shows that lambda phosphatase was able to dephosphorylate 4E-BP1 in extracts prepared at 48 hours (**Fig. 2A**, lanes 2 and 5 vs. lanes 1 and 4), in a process prevented by NaF (lanes 3 and 6 vs, lanes 2 and 5). 4E-BP1 was dephosphorylated in cells incubated with KU0063794, (lanes 7–9) and was not affected further by the purified phosphatase. Further analysis showed that KU0063794 treatment of cells reduced the phosphorylation of Thr37/46, Ser65 and Thr70 (**Fig. 2B**). In extracts prepared in the absence or presence of RAD001, 4E-BP1 phosphorylation of Thr37/46 remained resistant to lambda phosphatase treatment. These data confirm that the modification of 4E-BP1 observed during differentiation represents discrete phosphorylation.

### The ATM inhibitor, KU55399, does not affect phosphorylation of 4E-BP1 during myogenic differentiation

**Figure 3.**
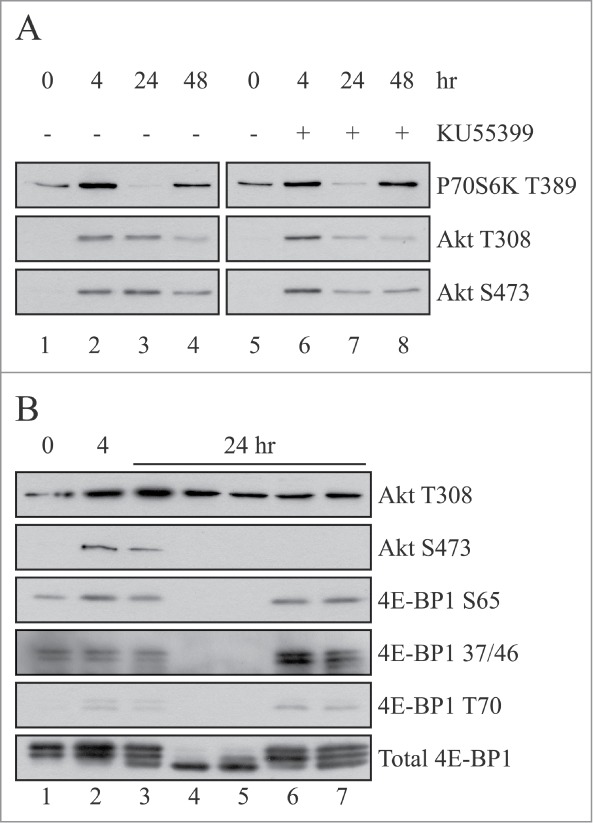
The ATM inhibitor, KU55399, does not affect phosphorylation of 4E-BP1. (**A**) Cells were grown to confluency and induced to differentiate by serum withdrawal for the times indicated in the absence (lanes 1–5) or presence of 10 µM KU55399 (lanes 6–8). Extracts were prepared as described in Materials and Methods and aliquots containing 10 µg total protein resolved by SDS-PAGE and proteins visualized by Western blotting using the antisera shown. (**B**) Cells were induced to differentiate by serum withdrawal for 0 (lane 1), 4 (lane 2) or 24 hours (lanes 3–7) in the absence (lanes 1,2) or presence of 100 nM RAD001 (lane 3), 100 nM Torin 1 (lane 4), 10 µM KU0063794 (lane 5), 10 µM KU55399 (lane 6) or 10 µM KU55399 and 100 nM RAD001 (lane 7). Aliquots containing 10 µg total protein were resolved by SDS-PAGE and proteins visualized by Western blotting using the antisera shown.

The work described above shows that the PI3-kinase inhibitor, LY294002, could partially inhibit phosphorylation of 4E-BP1 in C2C12 cells, similar to that seen with RAD001. Published work has suggested that insulin-induced phosphorylation of 4E-BP1 on Ser111 occurs in an Ataxia-Telangiectasia Mutated kinase (ATM)-dependent manner.^32^ To address whether ATM has a role in the RAD001-resistant phosphorylation of 4E-BP1 during differentiation of C2C12 myoblasts, we have used an inhibitor of ATM, KU55933.^33,34^ **Figure 3A** shows that KU55933 has no effect on the biphasic activation of p70S6K (as monitored by phosphorylation at Thr389) or the phosphorylation of Akt at Thr308. However, KU55933 was able to reduce Akt Ser473 phosphorylation at later times suggesting an inhibition of mTORC2 (lanes 7 and 8 vs. lanes 3 and 4). We have also directly compared the effects of inhibition of ATM on the phosphorylation of 4E-BP1 with those of RAD001, Torin 1 and KU0063794; unfortunately, no phospho-specific serum exists for the Ser111 site. **Figure 3B** shows that incubation of cells with KU55933 has a similar effect on 4E-BP1 phosphorylation as seen with RAD001 (lane 6 vs. lane 3). Addition of both KU55933 and RAD001 to cells did not further influence 4E-BP1 phosphorylation (lane 7 vs. lanes 6 and 3). RAD001 did however promote the phosphorylation of Akt Thr308 and reduce phosphorylation at Ser473. These findings are consistent with published data,^35-37^ possibly reflecting loss of inhibition of feedback signaling to IRS1 from p70S6K^37^ and inhibition of mTORC2.^35,36^ In contrast, both Torin 1 (lane 4) and KU0063794 (lane 5) completely inhibited 4E-BP1 and Akt phosphorylation at 24 hours incubation.

### Phosphorylation of 4E-BP1 is unaffected by siRNA-mediated depletion of raptor or rictor

**Figure 4.**
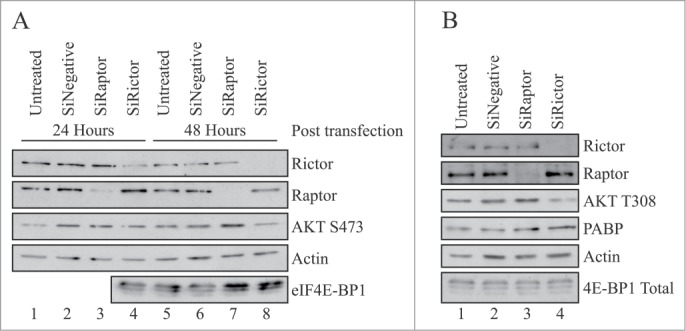
Phosphorylation of Akt Ser473 is stimulated siRNA-mediated depletion of raptor. (**A**) C2C12 cells were untransfected (lanes 1,5) or transfected with 9.4 nM Ambion Silencer pro-sequence siRNAs specific to either raptor (lanes 3,7), rictor (lanes 4,8) or a scrambled control siRNA (lanes 2,6), as described in Materials and Methods. After 24 hours, the medium was changed and cells were incubated in DM for the times indicated. Extracts were prepared and aliquots containing 10 µg total protein were resolved by SDS-PAGE for and proteins visualized by Western blotting using the antisera shown. (**B**) Cells were treated as described in Panel A and incubated in DM for 48 hours. Extracts were prepared and aliquots containing 10 µg total protein were resolved by SDS-PAGE for and proteins visualized by Western blotting using the antisera shown. Results are shown from a single experiment but similar data were obtained in at least 3 independent experiments. Depletion of raptor was determined at 95% +/−3% (S.D, n = 3).

To further examine the role for mTOR in the regulation of differentiation in C2C12 cells, we have ablated the expression of raptor and/or rictor by >95% using siRNAs (see Materials and Methods). After transfection, cells were harvested at different times and the level of raptor and rictor mRNA and protein depletion analyzed using qRT-PCR and Western blotting, respectively (**Figs. S2A** and **B**). Consistent with published data,^11,12^ depletion of raptor delayed, but did not prevent myogenic differentiation while depletion of rictor had little effect or slightly stimulated the process (data not shown) **Figures S2A** and **B** show that, relative to untreated cells, mRNA and protein for both raptor and rictor were depleted by >95% within 48 hours of transfection. In subsequent experiments, cells were incubated for 96 hours to allow for 48 hours in differentiation medium before extracts were prepared. SDS-PAGE analysis of extracts showed that depletion of raptor provoked an increase in the phosphorylation of Akt Ser473 (**Fig. 4A**, lane 7 vs, lanes 5 and 6) and to a lesser extent, AktThr308 (**Fig. 4B**, lane 3 vs lane 2). This was not evident at 24 hours (**Fig. 4A**, lanes 3 and vs, lanes 1 and 2) or following depletion of rictor at either time (lanes 4 and 8 vs. lanes 3 and 7). However, although depletion of rictor did not affect 4E-BP1 or Akt Ser473 phosphorylation, it did cause a reduction in Akt Thr308 phosphorylation at later times (**Fig. 4B**, lane 4 vs lane 2). Surprisingly, 4E-BP1 phosphorylation was unaffected by extensive raptor protein depletion at any time analyzed (**Figs. 4A** and **B**).

**Figure 5.**
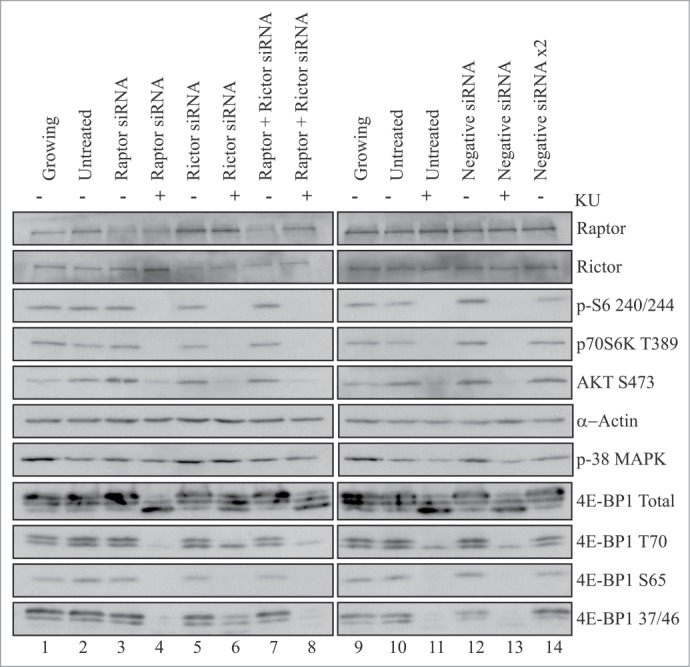
Phosphorylation of 4E-BP1 is unaffected by siRNA-mediated depletion of raptor or rictor in C2C12 cells. C2C12 cells were untransfected (lanes 1,2,9,10,11) or transfected with Ambion Silencer pro-sequence siRNAs specific to either raptor (lanes 3,4), rictor (lanes 5,6), both rictor and raptor (lanes 7,8), or appropriate scrambled control siRNAs (lanes 12–14), as indicated. After 24 hours, cells were either left in the original medium (lanes 1,9) or the medium changed to DM (lanes 2–8 and lanes 10–14) for 48 hours, in the absence (lanes 1–3, 5,7,9,10,12,14) or presence of 10 µM KU0063794 (lanes 4,6,8,11,13). Extracts were prepared and aliquots containing 10 µg total protein were resolved by SDS-PAGE for and proteins visualized by Western blotting using the antisera shown. Results are shown from a single experiment but similar data were obtained in at least 3 independent experiments. Depletion of raptor was determined at 95% +/−3% (S.D, n = 3).

To investigate this further, cells were incubated with a scrambled siRNA or depleted of either raptor, rictor or both proteins for 24 hours and cells subsequently allowed to differentiate for 48 hours in the absence or presence of KU0063794 (**Fig. 5**). Here we show that following depletion of raptor with siRNA (lane 3 vs lanes 2 and 12), there was no effect on p70S6K or rpS6 phosphorylation at 48 hours of differentiation. Similar findings were reported by Shu and Houghton (2009) who depleted C2C12 cells using lentivirus vectors.^12^ Depletion of raptor resulted in an increase in phosphorylation of Akt Ser473; this could not be attributed to activation of p38MAPK (**Fig. 5**). Again depletion of raptor had no effect on 4E-BP1 phosphorylation under these conditions. However, although the addition of KU0063794 decreased the efficiency of siRNA-mediated raptor depletion (lane 4 vs., lanes 3 and 2), it did prevent p70S6K activation, rpS6, 4E-BP1 and Akt Ser473 phosphorylation (lanes 4 and 13 vs, lane 3). Depletion of rictor using siRNA (lane 5 vs lanes 2 and 12) had no effect on p70S6K activity, S6 phosphorylation, Akt Ser473 phosphorylation or p38MAPK activity. Again there were no effects on 4E-BP1 phosphorylation but incubation of cells in the presence of KU0063794 resulted in inhibition of p70S6K, S6 phosphorylation, 4E-BP1 phosphorylation and Akt Ser473 (lanes 6 and 13 vs, lane 5). Depletion of both raptor and rictor (lane 7 vs. lanes 2 and 14) showed effects similar to depletion of rictor alone.

Considering that the mTORC1 complex is known to control 4E-BP1 phosphorylation during the upregulation of growth in a number of cell systems, we were somewhat surprised to find that extensive depletion of raptor had no effect on the KU0063794-sensitive phosphorylation of 4E-BP1 in C2C12 cells. Therefore, we used the same siRNAs to ablate raptor expression in murine NIH3T3 cells to see whether effects were cell-specific. **Figures S3A** and **B** show that ablation of either raptor or rictor did not affect total levels of 4E-BP1 in growing cells or in cells starved and then refed with 20% serum for 30 minutes. In contrast to C2C12 cells, decreased expression of raptor in NIH3T3 cells did not provoke an increase in the phosphorylation of Akt Ser473 (**Fig. S3B**), but rather increased Akt Thr308 phosphorylation, probably reflecting loss of inhibition of feedback signaling to IRS1 from p70S6K.^37^ In serum-starved cells, depletion of raptor (**Fig. S3A**, lane 9 vs lane 6) led to decreased AktThr308 and p70S6K phosphorylation, and partial inhibition of ERK phosphorylation. However, depletion of raptor seemed to protect 4E-BP1 phosphorylation from the effects of serum starvation. On the addition of serum there was an increase in ERK and total 4E-BP1 phosphorylation; consistent with published findings,^17-19^ raptor depletion prevented activation of p70S6K in NIH 3T3 cells (**Fig. S3A**, lane 12 vs. lane 9). To determine whether ERK activity was responsible for 4E-BP1 phosphorylation here, we repeated experiments in the absence or presence of PD184352, a characterized inhibitor of ERK.^38^ As shown in **Figure S3C**, inhibition of ERK did not prevent phosphorylation of 4E-BP1 in refed cells, either in the presence or absence of raptor. Ablation of rictor (lane 10 vs lane 6) led to increased p70S6K activity possibly reflecting increased expression of raptor.

## Discussion

The differentiation of skeletal muscle cells involves their exit from the cells cycle, changes in activity of an array of signaling pathways, altered gene expression, and cell fusion to form multinucleated myotubes.^1,2^ Previous work has shown a role for mTOR in this response^9-12,26^ but the role of 4E-BP1 phosphorylation remains elusive. Current models suggest that hyper-phosphorylated 4E-BP1 is released from eIF4E to allow for cap-dependent translation.^5,14^ During myogenic differentiation, this would promote the regulated recruitment of specific mRNAs into active eIF4F complexes, disassembly of stored mRNA from granules and the release of small, inhibitory RNAs (miRs) which prevent translation of the associated mRNAs.^5,17,22-25^

In the present study we show that myogenic differentiation promotes the hyperphosphorylation of 4E-BP1 and activation of p70S6K in C2C12 cells. Similar findings with 4E-BP1 have been reported for human megakaryocyte cells undergoing differentiation.^39^ **Figure 1** shows that inhibition of mTORC1 with RAD001 inhibited the phosphorylation of p70S6K and rpS6 and delayed differentiation (data not shown), but did not totally prevent the phosphorylation of 4E-BP1 on Thr37/46, Ser65 or Thr70. Phosphorylation of 4E-BP1 was prevented by KU0063794, a kinase inhibitor of mTORC1, similar to reports in HEK293 cells.^40^ We confirmed that 4E-BP1 modification recovered in extracts from RAD001-treated cells reflected protein phosphorylation as this was sensitive to lambda phosphatase *in vitro* (**Fig. 2** and **Fig. S1**).

The nature of the RAD001-resistant kinase responsible for 4E-BP1 phosphorylation here is unknown. However, one potential candidate is the Ataxia-Telangiectasia Mutated kinase, ATM.^32^ ATM kinase belongs to the PI3-kinase superfamily and cross-inhibition within this family is a common problem when using kinase inhibitors.^33^ In myotubes with shRNA-mediated ATM knockdown, cells showed decreased levels of Insulin-like growth factor 1-mediated phosphorylation of Akt Ser473, Akt Thr308 and p70S6 kinase activity.^41^ In addition, KU55933^32,33^ has been shown to prevent the stimulation of p70S6K phosphorylation in C2C12 myotubes exposed to IGF-1. To determine any potential role for ATM in the maintenance of 4E-BP1 phosphorylation when mTORC1 was inhibited, we used KU55933.^32,33^ **Figure 3** shows that ATM was not involved in the phosphorylation of 4E-BP1 during differentiation. Furthermore, as the PI3-K, Vps34, has been identified as a direct target of KU55933, our study also suggests that this PI3-K plays no role in controlling mTORC1 during myogenic differentiation.^34^ The Pim family kinases have also been shown to function in a rapamycin-insensitive way to mediate the phosphorylation of 4E-BP1 in AML cells.^42^ To investigate whether Pim kinases have a role in 4E-BP1 phosphorylation in C2C12 cells, we used the Pim kinase inhibitor, SGI-1776.^42,43^ In C2C12 cells, SGI-1776 had no effect of myogenic differentiation or the phosphorylation of 4E-BP1 (data not shown).

As KU0063794 has the ability to inhibit mTORC1, mTORC2, phosphorylation of 4E-BP1, p70S6K, rpS6, protein synthesis and differentiation, we explored mTORC signaling further using siRNA-mediated ablation of raptor or rictor to specifically target these signaling nodes.^16,18-20^

**Figures 4** and **S2** show that depletion of raptor was effective in these cells, it allowed cell fusion but delayed differentiation (data not shown). Relative to untreated cells, >95% depletion of raptor provoked an increase in the phosphorylation of Akt Ser473 (**Fig. 4A**) with no effect on 4E-BP1 phosphorylation (**Fig. 5**). Previous work has shown that during cells proliferation, disruption of raptor prevented mTORC1 signaling to p70S6K and 4E-BP1.^16,18^ Furthermore, skeletal muscle-specific ablation of raptor, but not rictor resulted in muscle dystrophy.^52^ mTORC1 disruption using raptor siRNA or RAD001 also failed to inhibit 4E-BP1 phosphorylation in AML cells.^43^ In megakaryopoiesis, the mTOR/rictor complex affects megakaryopoiesis by regulating nuclear division and subsequent cell cycle progression, whereas raptor signaling protects cells from autophagic cell death.^44^ Published work suggests that downregulation of rictor in C2C12 cells can inhibit mTORC2 signaling without inhibiting mTORC1; this prevented phosphorylation of Akt on Ser473 and stimulated protein synthesis.^12^ Our data show that extensive depletion of raptor had no effect on p70S6K or rpS6 phosphorylation during myogenic differentiation. However, phosphorylation of these proteins was still sensitive to the mTORC1/2 inhibitor, KU0063794. PRAS40 is unlikely have a role here as depletion of PRAS40 does not alter the phosphorylation of 4E-BP1 or p70S6K in either myoblasts or myotubes.^45,46^ As predicted, depletion of rictor by >95% had no effect on p70S6K, rpS6 or 4E-BP1 phosphorylation; depletion of both raptor and rictor showed effects similar to depletion of rictor alone. It seems very unlikely that the residual level of available raptor or rictor was sufficient to allow mTORC1/2 signaling toward 4E-BP1. SW620 colorectal cancer cells also show resistance to mTORC1-inhibiton with T37/46 phosphorylation of 4E-BP1 maintained in the presence of mTOR kinase inhibitors.^47^ As mTOR was catalytically inhibited (as indicated by the attenuation of S6K1 T389 and AKT S473 phosphorylation, the maintenance of 4E-BP1 phosphorylation likely reflects an mTOR-independent mechanism and not the disruption of the mTORC1 complex which can occur when high concentrations of inhibitors are used.^48^ The former is supported by the finding that raptor siRNAs did not affect 4E-BP1 phosphorylation in SW620 cells even though they effectively blocked S6K1 phosphorylation.^47^

Previous work has shown that the mTORC1 pathway is required for both differentiation and hypertrophy of C2C12 cells but p70S6K1 and 4E-BP1 are not required for the myogenic signaling of mTOR.^49^ The authors suggested that an acute mTOR-dependent signaling mechanism is essential for skeletal muscle differentiation, with increased transcription of negative regulators of mTORC1 such as PTEN, AMPKβ, or TSC2 up-regulated later during differentiation.^50^ However, we see no evidence for changes in the level of TSC2 protein or activation of AMPK in our study (data not shown). mTORC1 may be focused on modulating levels of autophagy,^7,13^ while mTORC2 signaling modulates the actin cytoskeleton and the down-regulation of the Rho associated kinase (ROCK) required for myogenesis.^12^ On the other hand, the requirement for acute mTOR signaling might reflect regulated changes in mitochondrial biogenesis and function, events required for myogenesis.^51^ Translation of a subset of mRNAs encoding nuclear-encoded, mitochondria-related proteins has been shown to be selectively suppressed following ablation of mTORC1 but not mTORC2^51^; decreased mTORC1 activity decreased mitochondrial function and ATP production. However, *in vivo* work indicates that raptor is critical in the longer term for muscle function and prolonged survival^52^; ablation of rictor had no affect on muscle function or morphology.

The question remains as to what links mTOR kinase with muscle cell hypertrophy. One possible link is eIF3; recent work^53^ has suggested that a conserved TOS motif in eIF3f allows it to interact with the mTOR/raptor complex, which phosphorylates S6K1^17^ and regulates downstream effectors of mTOR and cap-dependent translation initiation.^17,53^ Ablation of eIF3f in muscle cells prevented mTORC1 activity, phosphorylation of p70S6K1, rpS6, 4E-BP1 and myogenic differentiation. However, our data suggest that raptor, which recruits proteins with a TOS motif to mTOR kinase, is not required for the phosphorylation of p70S6K or 4E-BP1 in our cells, making this an unlikely explanation in C2C12 cells. Recent work from the Sonenberg group provides another possible explanation, which also presumes a central role for eIF3. This recent study shows an mTORC1-dependent interaction of PABP-interacting protein 1 (Paip1) with eIF3g,^54^ promoting rates of translation. The Paip1-eIF3 interaction was impaired by rapamycin and a rapalog but stimulated by p70S6K1. This work also showed that p70S6K1 interacts with, and phosphorylates eIF3f, leading the authors to suggest that p70S6K1 phosphorylates eIF3 to stimulate the Paip1-eIF3 interaction and promotes translation initiation.^53,54^ Whether mTOR or p70S6K directly phosphorylates eIF3f in C2C12 cells or the effects of ablation of eIF3f on differentiation are unknown at this time.

## Materials and Methods

### Chemicals and biochemicals

Materials for tissue culture were from Invitrogen, fetal calf serum was from Biosera, LY294002, microcystin LR and anti-actin antibody were from Sigma. RAD001^27^ was a gift from Novartis (Basel, Switzerland), Torin 1 was from the Sabatini lab,^13^ and KU0063794 and KU55933 were from Tocris. Unless otherwise stated, antisera to rictor, raptor, phospho-p70S6K (T389), phospho-S6 (S240/244), phospho-ERK (T202/Y204), phospho-p38MAPK (T180/Y182), phospho-Akt (T308 or S473), total 4E-BP1, phospho-4E-BP1 (T36/T37, S65 or T70) and antisera to total proteins were from Cell Signaling Technology. Antisera specific to eIF4GI and PABP were generated in-house.^28-30^ Immobilon PVDF was from GE Healthcare and unless otherwise stated, all other chemicals were from Sigma.

### Tissue culture

C2C12 cells, provided by the ECACC, were cultured in 10 cm plates in DMEM supplemented with 20% fetal calf serum at 37°C in a humidified atmosphere containing 5% CO_2_.^21^ NIH3T3 cells (passage #12), were maintained in DMEM supplemented with 20% fetal calf serum, as above. When required, C2C12 myoblasts were grown to 100% confluence in complete medium and induced to differentiate by serum withdrawal in the presence of 2% (v/v) horse serum, 10 µg/ml insulin and 10 µg/ml transferrin (DM). The medium was changed every 24 hours and if required, fresh inhibitors were added.

### Preparation of cell extracts

Following treatment, cells were isolated in a cooled centrifuge and washed briefly with 0.5 ml ice-cold PBS containing 2 mM benzamidine. Pellets were resuspended in 100 µl ice-cold Buffer A (20 mM MOPS(KOH), pH 7.2, 20 mM sodium fluoride, 1 µM microcystin, 75 mM KCl, 2 mM MgCl_2_, 2 mM benzamidine, 2 mM Na_3_VO_4_, complete protease inhibitor mix (-EDTA; Roche) and lysed by vortexing following the addition of 0.5% (v/v) Igepal and 0.5% (v/v) deoxycholate. Cell debris was removed by centrifugation in a microfuge for 5 min at 4°C^28-30^; a Bradford assay was used to determine protein content of extracts which were then frozen in liquid N_2_.

### Polyacrylamide gel electrophoresis (SDS-PAGE) and immunoblotting

Samples containing equal amounts of protein were resolved by PAGE (SDS-PAGE) and processed for Western blotting as described previously.^26,28-30^ Briefly, membranes were blocked using Tris-Buffered Saline (TBS)/0.5% (v/v) Tween containing 3% (w/v) BSA for 1 hour and incubated with antisera diluted in the same overnight at 4°C. Following washing in TBS-Tween, membranes were incubated with horseradish peroxidase-conjugated secondary antibody and signals developed using ECL.^28-30^

### Lambda phosphatase treatment of cell extracts

Aliquots of total cell extract (90 µg) were incubated with 200 units of lambda phosphatase (NEB) using the manufacturer’s conditions and buffers, in a final volume of 100 µl, in the presence of MnCl_2_. Incubations were carried out for the times indicated, in the absence or presence of 50 mM NaF; aliquots containing 10 µg extract protein were then removed into SDS-PAGE sample buffer and the phosphorylation of 4E-BP1 visualized by Western blotting.

### siRNA treatment of C2C12 cells

C2C12 cells (5 × 10^4^) plated in 6 well plates were grown to confluency and transfected with 9.4 nM Ambion Silencer pro-sequence siRNAs (Life Technologies) specific to either murine rictor, raptor or both together (or scrambled control) using Lipofectamine mRNAi Max. After 24 hours, the medium was changed to DMEM in the presence of 2% (v/v) horse serum, 10 µg/ml insulin and 10 µg/ml transferrin and cells incubated for the times indicated in the figure legends.

### siRNA treatment of NIH3T3 cells

Cells (1 × 10^5^) were plated in 6 well plates, grown for 24 hours and then transfected using Lipofectamine mRNAi Max with 9.4 nM Ambion Silencer pro-sequence siRNAs and cells were incubated for 48 hours before harvesting.

### Quantitative RT-PCR

RNA was extracted from cell extracts using an RNA easy mini-kit (Qiagen, UK) as per manufacturer’s instructions. RNA concentration was then quantified using a Nanodrop and 1µg of RNA was used for cDNA synthesis using the Promega ImpromII kit. The SYBR real-time PCR system (Kapa Biosystems) was used to quantify transcript abundance for genes of interest and 18S rRNA was used as a control. Template equivalent to 5 ng of RNA in cDNA library per reaction was added to each 20 μl reaction with a final primer concentration of 200 nM per reaction. Crossing thresholds were determined using MxPro software (Agilent), and fold-difference in RNA quantity was calculated using the relative quantification method (2^−ΔΔct^).
